# Radiomics for Detecting Metaplastic Histology in Triple-Negative Breast Cancer: A Step Towards Personalized Therapy

**DOI:** 10.3390/bioengineering12090973

**Published:** 2025-09-12

**Authors:** Rana Gunoz Comert, Gorkem Durak, Ravza Yilmaz, Halil Ertugrul Aktas, Zeynep Tuz, Hongyi Pan, Jun Zeng, Aysel Bayram, Baran Mollavelioglu, Sukru Mehmet Erturk, Ulas Bagci

**Affiliations:** 1 Department of Radiology, Istanbul Faculty of Medicine, Istanbul University, Istanbul 34093, Turkey; ravzaylmz@gmail.com (R.Y.); zeyneptuz@istanbul.edu.tr (Z.T.); smerturk@istanbul.edu.tr (S.M.E.); 2 Machine & Hybrid Intelligence Lab, Department of Radiology, Northwestern University, Chicago, IL 60611, USA; gorkem.durak@northwestern.edu (G.D.); halilertugrul.aktas@northwestern.edu (H.E.A.); hongyi.pan@northwestern.edu (H.P.); zeng.cqupt@gmail.com (J.Z.); ulas.bagci@northwestern.edu (U.B.); 3 Department of Pathology, Istanbul Faculty of Medicine, Istanbul University, Istanbul 34093, Turkey; aysel.bayram@istanbul.edu.tr; 4 Department of General Surgery, Istanbul Faculty of Medicine, Istanbul University, Istanbul 34093, Turkey; baran.mollavelioglu@istanbul.edu.tr

**Keywords:** metaplastic breast cancer, triple-negative breast cancer, radiomics, machine learning, Artificial Intelligence

## Abstract

This study aims to develop and validate a multisequence MRI-based radiomics approach for distinguishing metaplastic breast cancer (MBC) from non-metaplastic triple-negative breast cancer (TNBC) at the initial diagnosis, which could facilitate optimal treatment selection. In this retrospective study, we analyzed 105 patients (27 MBC, 78 non-metaplastic TNBC) who underwent standardized breast magnetic resonance imaging (MRI), which included T1-weighted contrast-enhanced (T1W-CE) and short-tau inversion recovery (STIR) sequences. Two radiologists performed ground truth lesion segmentation, verified by a senior radiologist. We extracted 214 radiomic features (using PyRadiomics) and used least absolute shrinkage and selection operator (LASSO) regression for feature selection. Seven machine learning classifiers were thoroughly evaluated using five-fold cross-validation, with performance assessed through ROC analysis and accuracy metrics. The combined T1W-CE and STIR analysis demonstrated superior diagnostic performance for distinguishing MBC from non-metaplastic TNBC (AUC = 0.845; accuracy = 81%) compared with either sequence alone (T1W only AUC = 0.805; accuracy = 80%; STIR only AUC:0.768; accuracy = 77%). Multisequence MRI radiomics can reliably distinguish between MBC and TNBC at the time of initial diagnosis. This could potentially facilitate the selection of more appropriate treatments and help avoid ineffective chemotherapy for MBC patients.

## 1. Introduction

Metaplastic breast cancer (MBC) is a rare (<1%) yet aggressive subtype of invasive breast cancer associated with a significantly poorer prognosis compared to non-metaplastic triple-negative breast cancer (TNBC) [1,2,3]. The five-year survival rate for non-metaplastic TNBC is approximately 80%, whereas the survival rate for MBC varies from approximately 30% to 70% [4,5]. Moreover, MBC cases have a higher risk of recurrence during follow-up, with 50% of patients experiencing local or distant metastases within five years of diagnosis [1]. The diagnostic challenge arises from a paradoxical similarity in molecular profiles: both MBC and standard TNBC exhibit a triple-negative hormone receptor pattern characterized by the absence of estrogen receptor (ER), progesterone receptor (PR), and human epidermal growth factor receptor 2 (HER2) expression [1,2,3,4]. This similarity contributes to the underdiagnosis of this distinct subtype, which may require different management strategies. To illustrate the complex relationship between these two entities, Table 1 comprehensively compares the general characteristics distinguishing MBC from non-metaplastic TNBC [1,2,3,5,6,7,8,9,10]. These differences, while subtle in some respects, have major implications for patient care and outcomes.

Core needle biopsies, which only sample a limited portion of the tumor, may miss these distinctive components, leading to a critical diagnostic blind spot. This sampling limitation often misclassifies MBC as standard TNBC, a seemingly minor error that carries significant therapeutic implications [11,12]. Perhaps most concerning is that this misdiagnosis frequently results in the initiation of neoadjuvant chemotherapy (NAC), a treatment approach that has consistently proven less effective in MBC cases. This diagnostic uncertainty creates a cascade of clinical consequences: patients receive suboptimal treatment while their disease may progress, valuable time is lost that could have been dedicated to more appropriate interventions, and the window for optimal therapeutic intervention is narrowed [13,14].

According to the latest National Comprehensive Cancer Network guidelines (v.6), NAC is the recommended preoperative treatment for non-metaplastic TNBC in clinical stages II and III [15]. It aims to downstage the disease and generally results in a favorable response. Achieving clinical downstaging facilitates breast-conserving surgery and allows for less invasive axillary surgery [15]. pCR rates in non-metaplastic TNBC range from 40% to 50%, while the overall response rate, including partial responses, is approximately 80% [16]. However, no specific treatment guidelines have been established for MBC [3,15]. While NAC is commonly used to treat many MBC patients, its effectiveness remains limited [14]. Patients often experience inadequate responses, disease progression, worsening clinical outcomes, significant time and financial losses, and treatment-related side effects. Research shows that approximately 50% of MBC patients treated with NAC may encounter inadequate responses or even disease progression, while the pCR rate is limited to between 11% and 17% [2,6,14]. This underscores a critical gap in the clinical management of this aggressive disease. Thus, it is crucial to identify these cases before initiating NAC treatment to prevent the side effects of overtreatment and ensure timely intervention.

There are no clearly defined radiological features that distinguish MBC from non-metaplastic TNBC using conventional imaging modalities, including mammography, ultrasound, and MRI evaluated by visual assessment, as both entities exhibit overlapping characteristics. Although an excisional pathological examination is considered the gold standard for diagnosis, it may not be feasible for every patient. Therefore, using more advanced techniques for image analysis is essential. Radiomics is an advanced image analysis method for extracting and analyzing quantitative features from medical images that are not visible to the naked eye [17]. It can help detect and characterize these subtle changes and potentially help to overcome challenges encountered in routine image evaluation and biopsy.

Our study directly addresses a critical gap in breast cancer diagnostics: the challenge of distinguishing MBC from non-metaplastic TNBC during the crucial initial evaluation stage. While conventional imaging features show overlap and biopsy methods often fail biopsy due to sampling errors that may cause metaplastic components to be missed, we propose that advanced imaging analysis could play a crucial role in early diagnosis of MBC [11,18]. This capability would enable clinicians to make more informed decisions about neoadjuvant chemotherapy, potentially sparing MBC patients from ineffective treatments while expediting more appropriate therapeutic interventions. This approach may represent a paradigm shift toward integrating standard imaging with precision oncology in managing aggressive breast cancer subtypes.

## 2. Methods

### 2.1. Study Design and Patient Cohort

This study was designed retrospectively. Our cohort consisted of 129 patients diagnosed between 2016 and 2024 at our institution, including 40 patients with confirmed metaplastic breast cancer and 89 patients with stage II and III non-metaplastic triple-negative breast cancer. All clinical and imaging data were fully anonymized in accordance with patient privacy protocols.

**Figure 1:**
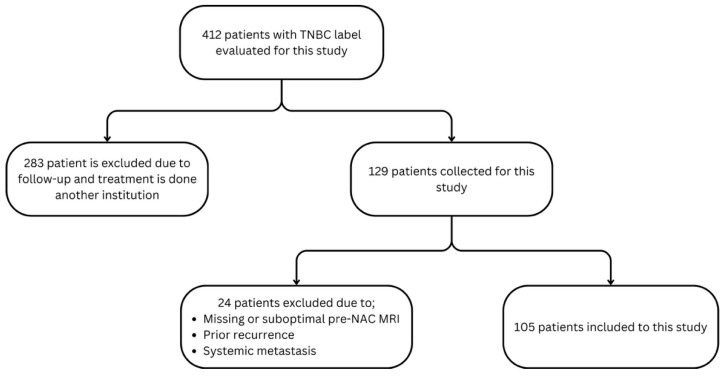
Flowchart of patient selection.

This study included patients with pathologically confirmed MBC and non-metaplastic TNBC who underwent pre-NAC breast MRI using standardized protocols at our institution. All biopsies, surgeries, and pathological assessments were performed at our institution. Exclusion criteria included MRI performed externally or after starting NAC, prior breast cancer treatment, and inadequate MRI quality to ensure consistency and minimize confounding factors (Figure 1).

### 2.2. MBC Cohort

**Figure 2:**
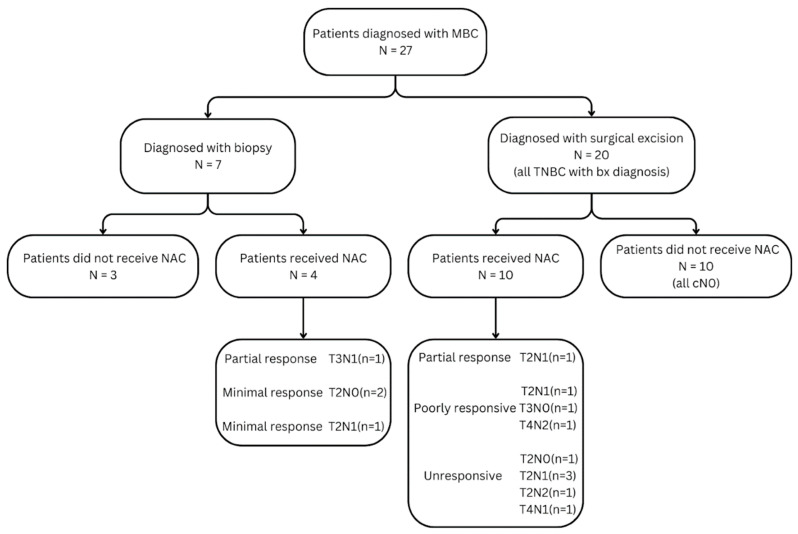
Clinical management diagram for patients diagnosed with MBC.

Among the 40 MBC patients, 13 were excluded due to missing or suboptimal pre-NAC MRI, prior recurrence, or systemic metastases (Figure 2). Diagnosis was made via core needle biopsy in 7 cases and postoperatively in 20. Hormone receptor analysis revealed one ER-positive, one HER2-positive, and one ER/PR low-positive case; the remainder were triple-negative (Table 2).

### 2.3. Non-Metaplastic TNBC Cohort

Among 89 patients diagnosed with non-metaplastic TNBC, 11 were excluded from the analysis for the following reasons: systemic metastases at diagnosis (*n* = 3), suboptimal MRI sequencing and quality (*n* = 5), and absence of pre-NAC imaging (*n* = 3). Consequently, 78 patients were included in this study, all categorized as stages II or III and having undergone NAC.

### 2.4. Neoadjuvant Chemotherapy Protocol

The NAC regimen included 4–8 cycles of doxorubicin–cyclophosphamide followed by paclitaxel. In the MBC group, two partial responders completed NAC, while others discontinued after four cycles of docetaxel–cyclophosphamide due to lack of radiologic response. The sole HER2-positive patient received anti-HER2 therapy. In the TNBC group, olaparib and pembrolizumab were added based on genetic profile and risk assessment.

### 2.5. Histopathologic Evaluation

Preoperative core needle biopsies and postoperative specimens were evaluated by two breast pathologists with 8 and 20 years of experience, respectively. Surgical management was determined based on the extent of the tumor, with breast-conserving surgery for unifocal or limited multifocal tumors and mastectomy for multicentric tumors. A sentinel lymph node biopsy (SLNB) was performed if the axillary lymph nodes showed a complete response to NAC; otherwise, axillary dissection was performed.

For patients receiving NAC, tumor bed sampling was determined by the size of the post-chemotherapy tumor bed (PCTB): one sample per centimeter for large PCTBs (>3 cm) and complete sampling for small PCTBs (<3 cm). In the absence of gross disease, a broad sampling of the affected quadrant was performed, guided by preoperative imaging. Microscopic examination revealed areas of stromal fibrosis consistent with regression, and regression rates were evaluated by reviewing all slides.

In this study, pathological response post-NAC was classified as: no response (no regression fibrosis), poor (<30% tumor regression), partial (30–99% regression or residual nodal metastasis), and complete (no residual tumor or nodal metastasis).

### 2.6. MR Image Acquisition

Dynamic contrast-enhanced breast MRI was performed using 1.5 T systems (Siemens Magnetom Aera, 18-channel; Philips Achieva, 16-channel). The MRI protocol began with acquiring axial (short tau inversion recovery) STIR images, axial T1W turbo spin-echo (TSE) images without fat suppression, and EPI2D diffusion-weighted imaging. Intravenous contrast (0.1 mmol/kg; Clariscan or Dotarem) was administered at 1 mL/s, followed by a 15 mL saline flush. Dynamic axial T1-weighted fat-suppressed imaging was acquired (1.5 mm slices, 60 s temporal resolution) over 5 min.

### 2.7. Ground-Truth Annotations and Inter-Observer Agreement

**Figure 3:**
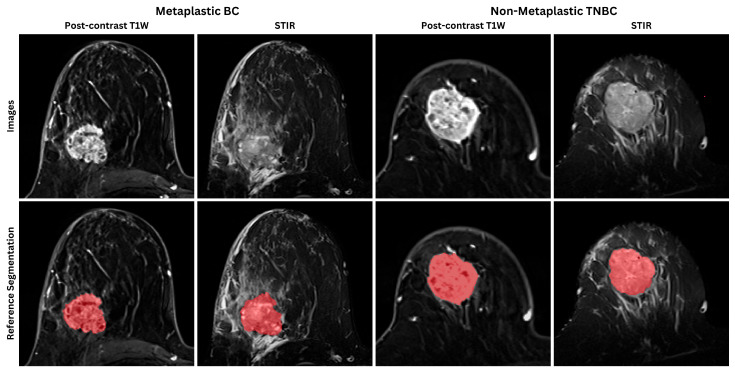
Representative T1W-CE and STIR images and reference segmentations of MBC and non-metaplastic TNBC subgroups. The red-marked areas show the segmented tumor lesion.

Post-contrast first-minute T1W and STIR MRI sequences were selected and converted to the Neuroimaging Informatics Technology Initiative (NIfTI) format for analysis using a 3D Slicer (Version 5.6.2) [19]. Two radiologists, with 7 and 10 years of experience, independently segmented the index lesions in T1-CE and STIR using the same software (Figure 3). An experienced breast radiologist with 20 years of experience reviewed the results to ensure accuracy and consistency. The consistency of the segmentations was evaluated by calculating the Dice Similarity Coefficient (DSC) and Hausdorff distance (HD95) to assess both interobserver and intraobserver agreement. Thirty MRI scans were randomly selected to evaluate interobserver agreement. For intraobserver agreement, the same radiologists repeated the segmentation on 20 randomly selected MRI scans a second time after a washout period of two weeks. The DSC for intraobserver agreement was 95%, with an HD95 of 5.73 mm. The interobserver DSC was 89%, with an HD95 of 8.32 mm.

### 2.8. Procedure for the Radiomics Analysis

Using PyRadiomics, we extracted a comprehensive set of 214 radiomic features including shape-based features (geometric properties such as volume, surface area, and sphericity), first-order statistical features (intensity histogram distribution characteristics), and second-order texture features. The texture features comprised Gray Level Co-occurrence Matrix (GLCM) features that capture spatial relationships between pixel intensities, Gray Level Run Length Matrix (GLRLM) features that measure consecutive pixels with the same intensity, Gray Level Size Zone Matrix (GLSZM) features that quantify homogeneous regions, Gray Level Dependence Matrix (GLDM) features, and Neighboring Gray Tone Difference Matrix (NGTDM) features that assess local intensity differences.

Our study employed a sophisticated feature selection strategy utilizing five-fold cross-validation to ensure robust model performance. Within each training fold, we implemented a two-stage feature filtering process. First, we conducted Pearson correlation analysis to identify and remove highly correlated features (correlation coefficient > 0.9) while simultaneously eliminating low-variance features (variance < 5 × 10^−4^) that would contribute minimal discriminative value [20]. Building upon this initial filtering, we applied LASSO regression to identify additional features that might be redundant or less informative for our classification task. We then developed an innovative approach to feature consistency: by tracking the frequency of feature selection across all five folds, we identified features that consistently appeared redundant in at least three folds. This frequency-based selection method systematically eliminated consistently redundant features from our original feature set, ensuring that our final model would rely on the most stable and informative imaging characteristics.

**Figure 4:**
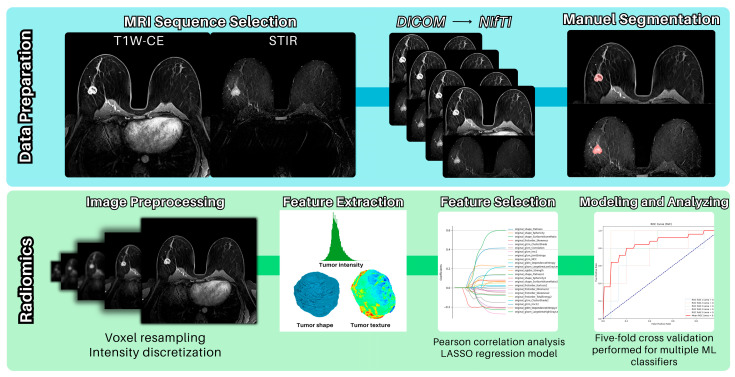
Overview of data preparation and radiomics analysis steps.

Using the optimized feature set, we trained multiple machine learning classifiers: Decision Tree (a tree-based model using binary splits), Random Forest (an ensemble of decision trees), K-Nearest Neighbors (distance-based classification), Support Vector Machine (SVM, finding optimal class-separating hyperplanes), Gaussian Naive Bayes (probabilistic Bayesian classifier), Logistic Regression (linear probabilistic model), and Gradient Boosting (sequential ensemble method) [21,22,23,24,25,26,27]. All implementations utilized Python (3.8.10.)’s scikit-learn library with five-fold cross-validation for performance evaluation. In more detail, the K-nearest Neighbors algorithm was applied with K = 11 neighbors, and the Random Forest method was implemented using 600 estimators. The SVM took an RBF kernel with C = 1.0, with gamma = ‘scale’. Figure 4 presents the overall steps of data preparation and radiomics analysis.

### 2.9. Statistical Analysis

Descriptive and comparative statistical analyses were performed for all study variables. Continuous variables were summarized as mean ± standard deviation and median [interquartile range]. Normality of distributions was assessed using the Shapiro–Wilk test. Between-group comparisons of continuous variables were performed using Welch’s *t*-test for normally distributed data and the Mann–Whitney U test for non-normally distributed data. Categorical variables were summarized as frequencies (n, %) and compared descriptively between groups. Two-sided *p*-values < 0.05 were considered statistically significant. All statistical analyses were conducted using SPSS (Statistical Package for the Social Sciences) software version 26 (IBM Corp., Armonk, NY, USA).

## 3. Results

Our final study cohort consisted entirely of female patients, with balanced representation from both cancer subtypes. The metaplastic breast cancer group included 27 patients with a median age of 45 years (range: 30–66 years), while the non-metaplastic triple-negative breast cancer group included 78 patients with a median age of 48 years (range: 27–81 years). Importantly, tumor characteristics were comparable across groups: MBC patients had tumors with a mean diameter of 39.5 mm and a median of 32 mm (range: 13–110 mm), closely paralleling the non-metaplastic TNBC group’s mean tumor diameter of 38.6 mm and median of 33 mm (range: 8–89 mm). This similarity in baseline tumor dimensions strengthens the validity of our comparative analysis by minimizing size-related bias in the imaging features.

For the MBC group, the diagnosis was made through core needle biopsy in 7 patients (25.9%), while the remaining 20 patients (74.1%) were diagnosed post-surgically. Of the 14 MBC patients who underwent NAC, 6 (42.9%) were classified as non-responders, 6 (42.9%) as poor responders, and 2 (14.2%) as partial responders (Figure 2). Among the 27 cases, twelve were undifferentiated type, six were of the matrix-producing type, nine were squamous type, and one was apocrine and spindle sarcomatous type, histologically (Table 2). Notably, two patients who achieved a partial response to NAC had matrix-producing histological subtypes.

The non-metaplastic TNBC group exhibited distinct patterns in treatment response that warrant careful analysis. All 78 patients in this cohort underwent a diagnostic biopsy followed by neoadjuvant chemotherapy, providing a robust dataset for evaluating treatment efficacy. The response patterns indicated a tendency toward positive outcomes: nearly half of the patients (48.7%, *n* = 38) achieved a complete response, while an additional 34.6% (*n* = 27) demonstrated a partial response. This collective 83.3% favorable response rate aligns with expected treatment outcomes for standard TNBC. However, a smaller subset of patients exhibited resistance to therapy: 14.1% (*n* = 11) showed a poor response, and 2.5% (*n* = 2) displayed no response. Interestingly, despite the small sample size, among the two non-responders, both tumors exhibited specific differentiation patterns, one with squamous features and the other with papillary characteristics, and were classified as grade 2 tumors. The poor responder group also presented intriguing histological patterns, with one case showing squamous differentiation while the remaining cases manifested as nonspecific invasive carcinoma (Table 2).

**Figure 5:**
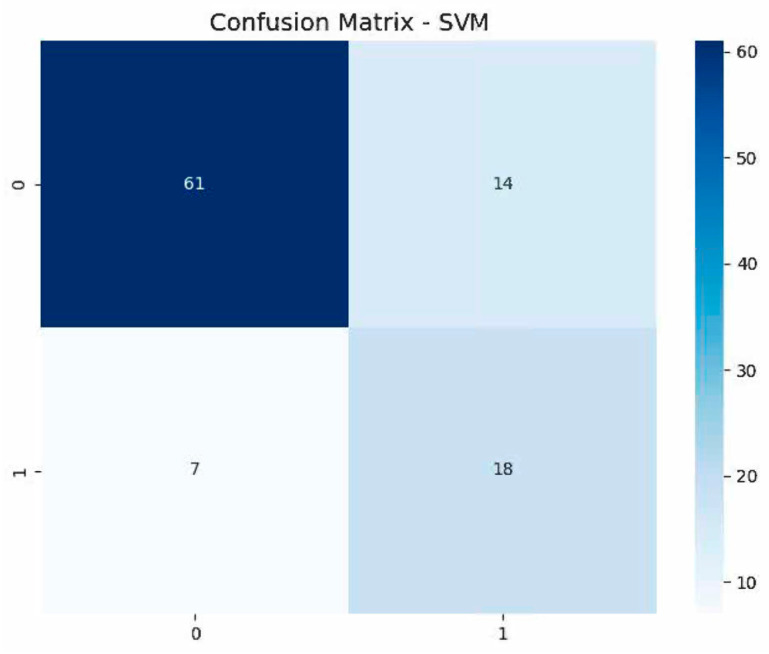
Confusion matrix for the SVM classifier using combined T1W-CE and STIR radiomics features. Confusion matrix shows 18 correctly identified MBC cases and 61 correctly identified TNBC cases out of the total cohort.

**Figure 6:**
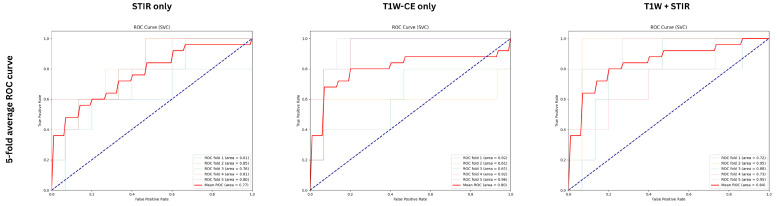
Representative 5-fold cross-validation average ROC curves for SVM classifier across T1W-CE and STIR analysis.

Our radiomics analysis demonstrated that multisequence imaging outperformed single-sequence methods in differentiating MBC from non-metaplastic TNBC. The combined assessment of STIR and T1W-CE sequences yielded the most comprehensive feature set, extracting 214 radiomic features. Through a rigorous feature selection process using LASSO, we identified 23 key features that proved to be the most informative for classification. This multisequence approach achieved the highest performance metrics, with a mean AUC of 0.845 and an accuracy of 81% (Table 3), showcasing the synergistic value of utilizing complementary imaging sequences. The detailed classification performance for the SVM classifier is illustrated in the confusion matrix (Figure 5), which shows 18 correctly identified MBC cases and 61 correctly identified TNBC cases out of the total cohort. While single-sequence analyses remained effective, they displayed slightly lower performance metrics. The T1W-CE sequence alone generated 107 radiomic features, of which 19 were selected as the most significant. This analysis achieved a mean AUC of 0.805 and an accuracy of 80% (Table 4). Similarly, the STIR-only analysis, which also produced 107 initial features but retained only 10 after LASSO selection, matched these performance metrics with a mean AUC of 0.768 and an accuracy of 77% (Table 5). The comparative ROC curves for the SVM classifier across all three approaches are illustrated in Figure 6.

## 4. Discussion

Our study demonstrates the potential of advanced radiomics analysis in addressing a critical clinical challenge: distinguishing MBC from non-metaplastic TNBC at the time of initial diagnosis. The differences in treatment responses between these groups, with 85.8% of MBC patients showing poor or no response to NAC compared to 83.4% of TNBC patients achieving complete or partial response, underscore the urgent need for accurate early identification. While our cohort size of 27 MBC cases may seem modest, it represents a substantial collection, given the rarity of MBC (affecting less than 1% of breast cancer patients), and provides sufficient statistical power for our analysis.

The strength of our findings lies in the robust performance across multiple analytical approaches. The combined analysis of T1W-CE and STIR sequences achieved promising results (AUC 0.845, accuracy 81%), while the single-sequence analyses showed reliable performance (T1W only AUC = 0.805, accuracy = 80%; STIR only AUC = 0.768, accuracy = 77%). This consistency across various imaging combinations highlights the stability of our radiomics approach. Our feature selection process, which identified 23 key features from an initial pool of 214 in the combined analysis, demonstrates the ability to capture meaningful imaging characteristics despite the limited sample size.

While radiomics is a widely used method for predicting various subgroups of breast cancer through MRI, to our knowledge, this is the first study in the literature that addresses this complex issue. Many studies focus on classifying breast cancer broadly or differentiating between TNBC and non-TNBC tumors. In a study conducted by Huang et al., the researchers analyzed 377 patients, dividing them into 202 for training, 87 for validation, and 88 for testing [28]. They successfully differentiated four tumor subtypes (Luminal A, Luminal B, HER2-enriched, and triple-negative) by extracting intra- and peri-tumoral features from dynamic contrast-enhanced sequences using radiomics analysis. In the sub-analysis of the study, the combined intra- and peri-tumoral model indicated an AUC of 0.718, while the full-fusion model indicated an AUC of 0.721 in differentiating 75 TNBC patients from 302 non-TNBC patients.

In a similar study by Yue et al. involving 516 patients, the highest average AUC performance for distinguishing all four molecular subtypes reached 0.8623. In a subanalysis of the same study, the AUC for differentiating 72 TNBC patients from 444 non-TNBC patients was 0.93 [29]. In another study by Ni et al., examining a four-class classification with 112 patients, the overall accuracy of the radiomics model under cross-validation was 82.1% [30]. All these studies utilized larger patient populations and tumor subtypes that are more easily radiologically distinguishable from each other than our study. However, our combined radiomics model performed well at distinguishing tumor subtypes, even though their radiological appearances were nearly identical.

Other studies suggest that the radiomics analysis of MRI images for breast cancer can benefit several other applications. For example, research on 163 TNBC patients assessed the treatment response to NAC, revealing that radiomics analysis using pre-treatment dynamic contrast-enhanced and DWI sequences achieved an impressive success rate of 80% [31]. Additionally, another study indicated that classifying TNBC cases based on NAC responses could reach an accuracy of 80% through clinicopathological modeling and MRI radiomics analysis [8]. These studies also demonstrated that MRI-based radiomics analysis for predicting response to NAC—incorporating dynamic sequences, DWI, T2W, and other sequences improves predictive accuracy [32].

Several aspects of our study design help mitigate potential limitations. First, our use of cross-validation strengthens the reliability of our results despite the modest sample size. Second, the inclusion of all available MBC cases from our institution over an eight-year period represents a realistic clinical scenario. Third, our cohort’s clear dissimilarity in treatment responses between MBC and TNBC patients aligns with previously reported patterns, suggesting that our patient population represents the broader clinical experience. Matrix-producing MBCs demonstrate a more favorable response to NAC than non-matrix-producing subtypes [2,3,6,14,33]. However, further subgroup analysis to differentiate between matrix-producing and non-matrix-producing carcinomas was not feasible in this study due to the limited sample size. Finally, a notable consideration in our study is the focused analysis of specific MRI sequences: post-contrast, first-minute T1W-CE, and STIR. While this targeted approach yielded promising results in differentiating MBC from non-metaplastic TNBC, we recognize that breast MRI offers a broader array of potential imaging biomarkers through additional sequences. The incorporation of diffusion-weighted imaging (DWI), dynamic contrast-enhanced series across multiple time points, and advanced quantitative parameters could potentially enhance the discriminative power of our radiomics approach.

## 5. Conclusions

A significant proportion of MBC cases are initially misclassified as non-metaplastic TNBC through biopsy, yet they often resist conventional NAC treatment. Advanced MRI radiomics analysis can help distinguish MBCs from non-metaplastic TNBC, thus aiding in selecting the optimal treatment strategy for patients. Our study demonstrates that advanced MRI radiomics analysis offers a powerful solution to this clinical dilemma. By achieving 81% accuracy through the combined analysis of T1W-CE and STIR sequences, our approach provides a robust, non-invasive method for the early diagnosis of these distinct cancer subtypes. This work represents a significant step toward personalized treatment planning in aggressive breast cancers, potentially sparing patients from ineffective therapies while accelerating appropriate interventions.

## Figures and Tables

**Table 1:** Comparison of general characteristics of MBC and non-metaplastic TNBC.

	Metaplastic Breast Cancer (TNBC)	Non-Metaplastic Triple-Negative Breast Cancer (TNBC)
**Frequency**	Rare (<1% of all breast cancers)	Accounts for 10–20% of all breast cancers
**Histology**	Heterogeneous, with epithelial and mesenchymal differentiation; may include spindle cells, squamous cells, or chondroid/osteoid elements	Homogeneous histology; lacks the diverse cellular differentiation seen in MBC
**Receptor Status**	Usually triple-negative (ER-, PR-, HER2-), though rare exceptions exist	Defined by triple-negative receptor status
**Aggressiveness**	Highly aggressive; disease-free survival is lower	An aggressive tumor profile
**Lymph Node Involvement**	Less common compared to non-metaplastic TNBC	More frequent compared to MBC
**Metastasis Pattern**	More likely to metastasize to lungs, bone, and distant sites; lower rates of lymphatic spread	Commonly metastasizes to lungs, brain, higher rate of lymphatic spread
**Prognosis**	Poorer prognosis than non-metaplastic TNBC due to treatment resistance; lower survival rates, especially in metastatic disease	Poor prognosis compared to hormone receptor-positive breast cancers, but slightly better than MBC
**Response to NAC**	Frequently resistant to standard NAC	Chemo-sensitive, partial and complete pathologic response rates were remarkably high
**Clinical Trials**	Heavily reliant on clinical trials due to lack of standardized therapies	Clinical trials also recommended but more established guidelines exist

**Table 2:** Clinicopathological characteristics of MBC and non-metaplastic TNBC groups.

Characteristics	Metaplastic Breast Cancer (MBC)	Non-Metaplastic Triple-Negative Breast Cancer (TNBC)	*p*-Value
**Initial patients**	40	89	
**Exclusions**	13 (lack of perioperative MRI, lack of pre-NAC imaging, suboptimal MRI sequencing, recurrence, or systemic metastases)	11 (systemic metastases: 3, suboptimal MRI sequencing: 5, lack of pre-NAC imaging: 3)	
**Patients analyzed**	27	78	
**Age**			0.090
Mean ± SD (years)	44.2 ± 10.1	48.2 ± 11.0	
Median (IQR) (years)	45 [37–48]	48 [40–55]	
**Tumor size**			0.560
Mean ± SD (mm)	39.5 ± 25.0	39.0 ± 18.3	
Median (IQR) (mm)	32 [26–42]	33 [28–48]	
**Hormone receptor profiles**	40% ER+ (1 case), HER2+ (1 case), 5% ER/5% PR+ (1 case), others TN	All TN	
**NAC response**	Non-responders: 6 (42.9%) Poor responders: 6 (42.9%) Partial responders: 2 (14.2%)	Complete response: 38 (48.7%) Partial response: 27 (34.6%) Poor response: 11 (14.1%) No response: 2 (2.5%)	
**Histological subtypes**	Undifferentiated *n* = 12 (44.4%), Matrix-producing *n* = 6 (22.2%), Squamous differentiation *n* = 9 (33.3%), Apocrine/spindle sarcomatous features *n* = 1 (3.7%)	Non-specific invasive carcinoma Squamous-differentiated invasive ductal carcinoma (1 case among poor responders) Papillary-differentiated invasive ductal carcinoma (1 case among non-responders)	

**Table 3:** 5-Fold Cross-Validation Performance of ML Models Using Post-Contrast T1W and STIR Images. This multisequence approach achieved the best performance AUC = 0.845, accuracy = 81%, demonstrating the advantages of combining sequences.

ML Model	AUC	Prec (%)	Sens (%)	Spec (%)	F1 (%)	Acc
Decision Tree	0.700	73.00	48.00	92.00	55.30	**0.81**
Random Forest	0.724	45.21	60.00	68.00	49.68	0.66
KNeighbors	0.641	53.56	48.00	80.00	49.54	0.72
SVM	**0.845**	59.33	72.00	81.33	64.12	0.79
GaussianNB	0.629	25.08	72.00	32.00	36.48	0.42
Logistic Regression	0.832	61.09	76.00	81.33	66.32	0.80
Gradient Boosting	0.811	59.39	60.00	81.33	56.99	0.76

AUC: Area Under The Curve, Prec: Precision, Sens: Sensitivity, Spec: Specivity, F1: F1 score, Acc: Accuracy.

**Table 4:** 5-Fold Cross-Validation Performance of ML Models Using Post-Contrast T1W-Only Images. T1W images alone analysis achieved a mean AUC of 0.805 and accuracy = 80%, performing slightly lower performance than the multisequence combined analysis.

ML Model	AUC	Prec (%)	Sens (%)	Spec (%)	F1 (%)	Acc
Decision Tree	0.687	59.76	56.00	81.33	53.22	0.75
Random Forest	0.663	39.17	52.00	65.33	42.56	0.62
KNeighbors	0.540	38.00	24.00	80.00	25.26	0.66
SVM	**0.805**	68.43	68.00	84.00	65.67	**0.80**
GaussianNB	0.467	17.45	56.00	26.67	26.58	0.34
Logistic Regression	0.779	60.50	68.00	81.33	62.05	0.78
Gradient Boosting	0.664	58.00	56.00	78.67	52.49	0.73

AUC: Area Under The Curve, Prec: Precision, Sens: Sensitivity, Spec: Specivity, F1: F1 score, Acc: Accuracy.

**Table 5:** 5-Fold Cross-Validation Performance of ML Models Using STIR-Only Images. STIR images alone analysis achieved a mean AUC of 0.768 and an accuracy of 77%, which was lower than the T1-only and multisequence-combined analyses.

ML Model	AUC	Prec (%)	Sens (%)	Spec (%)	F1 (%)	Acc
Decision Tree	0.680	58.00	56.00	80.00	50.93	0.74
Random Forest	0.713	44.96	60.00	72.00	49.23	0.69
KNeighbors	0.660	42.71	52.00	77.33	46.44	0.71
SVM	**0.768**	59.14	60.00	82.67	57.91	**0.77**
GaussianNB	0.555	25.92	60.00	42.67	36.14	0.47
Logistic Regression	0.757	55.24	52.00	78.67	47.06	0.72
Gradient Boosting	0.755	47.83	56.00	76.00	49.69	0.71

AUC: Area Under The Curve, Prec: Precision, Sens: Sensitivity, Spec: Specivity, F1: F1 score, Acc: Accuracy.

## Data Availability

The datasets generated and/or analysed during the current study are not publicly available due to ethical considerations/institutional policies but are available from the corresponding author on reasonable request.

## Abbreviations

The following abbreviations are used in this manuscript:

AUC	Area under the curve
DWI	Diffusion-weighted imaging
ER	Estrogen receptor
FDG	Fluorodeoxyglucose
HER2	Human epidermal growth factor receptor 2
MBC	Metaplastic breast cancer
MRI	Magnetic resonance imaging
NAC	Neoadjuvant chemotherapy
pCR	Pathological complete response
PCTB	Post-chemotherapy tumor bed
PET-CT	Positron emission tomography and computed tomography
PR	Progesterone receptor
SLNB	Sentinel lymph node biopsy
STIR	Short tau inversion recovery
SVM	Support Vector Machine
TNBC	Triple-negative breast cancer
